# *Legionella pneumophila* Effector Protein LegU1 Mediates the Ubiquitination of Endoplasmic Reticulum Chaperone BiP

**DOI:** 10.4014/jmb.2507.07056

**Published:** 2025-10-28

**Authors:** Beibei Zhao, Shiyu He, Minjun Huang, Yongjun Lu, Zhenhuang Ge

**Affiliations:** 1 School of Chemical Engineering, Xuzhou College of Industrial Technology, Xuzhou 221140, P.R. China; 2 Engineering Laboratory of High Efficiency and Comprehensive Utilization of Biochemical Resources, Xuzhou 221140, P.R. China; 3 School of Life Sciences, Sun Yat-sen University, Guangzhou 510275, P.R. China; 4 School of Medicine, Chongqing University, Chongqing 400030, P.R. China

**Keywords:** *Legionella pneumophila*, LegU1, BiP, ubiquitination

## Abstract

The facultative intracellular pathogen *Legionella pneumophila*, which causes Legionnaires' disease, translocates over 300 effector proteins into the host cell. By hijacking numerous host cellular signaling pathways, these effectors promote bacterial survival and growth. One of the effector proteins, LegU1, is a F-box containing protein that binds to the host cell protein Skp1 to form a Skp1-Cullin-F-box protein (SCF) complex conferred E3 ubiquitin ligase activity. However, the role of LegU1 during *L. pneumophila* infection is incompletely known. Here, we demonstrate that LegU1 participates in modulation of the host BiP, an important endoplasmic reticulum chaperone that functions as both a master regulator and target protein of the unfolded protein response (UPR) process, during *L. pneumophila* infection. Ectopically expressed LegU1 localizes to the endoplasmic reticulum (ER) through the region from the 88th to the 136th residue. Deletion of LegU1 increases the protein level of BiP during *L. pneumophila* infection. We finally indicate that LegU1 interacts with and mediates the ubiquitinational degradation of BiP. Altogether, our study identifies BiP as a new substrate of LegU1 and provides new insight into how *L. pneumophila* modulates the host UPR pathway during infection.

## Introduction

*Legionella pneumophila* is a Gram-negative bacterium and the causative agent of Legionnaires’ disease and Pontiac fever [1]. The ability of *L. pneumophila* to survive and replicate within human alveolar macrophages is key to its virulence. After phagocytosis, *L. pneumophila* remodels the phagosome into an endoplasmic reticulum (ER)-like compartment, known as the *Legionella*-containing vacuole (LCV), thereby sheltering itself from phago-lysosomal degradation and creating a specialized niche for replication [2]. The formation and maintenance of the LCV rely on the functional type IVB secretion system (T4BSS), from which numerous substrate proteins called effectors are delivered into the host cytoplasm during infection [3]. These effectors participate in the manipulation of multiple host cellular pathways to facilitate bacterial proliferation and survival *in vivo* [4]. Although over 330 effectors have been identified, only a few of them have been characterized in detail because of functional redundancy between effectors [5].

Ubiquitination is a crucial posttranslational modification of proteins that regulates numerous cellular processes, including protein homeostasis, immune response and vesicular trafficking [6]. The procedure of ubiquitination involves the covalent attachment of single or multiple ubiquitin molecules to target proteins, thereby affecting their localization, activity, conformation, stability and interaction network [6, 7] . In general, ubiquitination occurs through a cascade of reactions. First, free ubiquitin is activated by ubiquitin-activating enzymes (E1). The E1-linked ubiquitin is then transferred to ubiquitin-conjugating enzymes (E2). Finally, ubiquitin-protein ligases (E3) transfer ubiquitin from the E2s to the substrates by binding to both E2 enzymes and substrates [8]. Among hundreds of E3 enzymes, the Skp1-Cullin-F-box protein (SCF) E3 ubiquitin ligase is one of the best studied. In this ligase, the cullin subunit (usually CUL1) serves as a central structural framework that supports the assembly of other components. The C-terminal of CUL1 binds to the E2 enzyme via the small RING-box protein, RBX1. The N-terminal of CUL1 interacts with Skp1, which binds to the F-box domain of an interchangeable F-box protein. F-box proteins serve as the substrate-recognition modules of the SCF complex and thereby guide the ubiquitination of specific target proteins [9, 10].

Bacterial pathogens have been shown to evolve multiple mechanisms to manipulate the host ubiquitination system [11]. In *L. pneumophila*, several T4BSS effector proteins have been identified to exhibit E3 ubiquitin ligase activity [3]. For example, LubX is a U-box domain-containing E3 ligase and mediates poly-ubiquitination of host protein Clk1 [12]. Furthermore, LubX also ubiquitinates effector SidH, leading to its degradation [13]. SidC functions as an E3 ligase activity which is essential for mediating the recruitment of ER-derived elements and polyubiquitined proteins to the LCV [14]. *L. pneumophila* also encodes numerous F-box effectors. These effectors have been proven to function in infected cells as components of SCF E3 ligases [15].One of these proteins is LegU1, a 188 amino acid protein comprising an N-terminal F-box domain. A previous study reported that LegU1 binds the host cell protein Skp1 through its F-box domain, which leads to the formation of active SCF complex. *In vitro* ubiquitination assay has validated that SCF^LegU1^ directs the ubiquitination of the host chaperone protein BAT3 [16]. However, to date, the biological implication of LegU1 during *L. pneumophila* infection is still elusive.

In the present study, we aimed to explore the function of LegU1. We report here that deletion of LegU1 impairs the suppression of the upregulation of BiP during *L. pneumophila* infection. Further investigation demonstrate that LegU1 interacts with BiP and targets it for ubiquitination. Moreover, deletion of LegU1 decreased the ubiquitinational degradation of BiP in host cells during infection. Our study identifies a new substrate of LegU1 and one mechanism that how *L. pneumophila* modulates BiP during infection. Since BiP is considered as the key regulator and biomarker of the unfolded protein response (UPR), a highly conserved pathway in eukaryotes to restore cellular homeostasis following physiological stress exerted on the ER [17], and *L. pneumophila* has the ability to suppress certain arms of the UPR [18], our data provide a new insight to understand the strategies used by *L. pneumophila* to modulate the UPR during infection.

## Materials and Methods

### Bacterial Culture

The bacterial strains and plasmid vectors utilized in this study are provided in Table S1. Bacterial cultures of *L. pneumophila* were maintained on buffered charcoal yeast extract (BCYE) agar medium, or in liquid culture using N-(2-acetamido)-2-aminoethanesul-fonic acid (ACES)-buffered yeast extract (AYE) medium. *Escherichia coli* strains were cultivated using Luria-Bertani (LB) broth or agar. Antibiotics were incorporated into the medium when necessary: chloramphenicol at 30 μg/ml, ampicillin at 100 μg/ml, or kanamycin at 100 μg/ml [19].

### Mutant Construction and Complementation Assay

The Δ*legU1* mutant *L. pneumophila* strain was constructed as described before [19]. To delete *legU1*, the upstream and downstream of flanking sequences of *legU1* were amplified by PCR using following primers: P_XU1-F1_ (CCGCTCGAGGGATACAAGATAAACGTATAGCAGT), P_XU1-R1_ (AATTAAATAATTTAGGTTATTCACT TTGGGGTCTTGGCCCG), P_XU1-F2_ (CGGGCCAAGACCCCAAAGTGAATAACCTAAATTATTTAATT) and P_XU1-R2_ (CGGGATCCCCGTCGGTAAAACCATAG). The PCR products were used as template DNA for a subsequent PCR step, with primers P_XU1-F1_ and P_XU1-R2_. The resulting product was cloned into the pBRDX vector to generate pBRΔ*legU1* [20]. This plasmid was subsequently electroporated into the wild-type strain, transformants were selected on BCYE agar containing chloramphenicol. Colonies were patched, cultured in AYE liquid medium and plated on BCYE supplemented with metronidazole. After incubation at 37°C for approximately three days, clones that had lost the suicide vector were identified. The Δ*legU1* strains were confirmed by PCR and DNA sequencing. To construct plasmids that expressed *legU1* in *L. pneumophila*, the promoter of *mip* and 3 × Flag-tagged *legU1* were fused and cloned into pJB908 [21]. The following primers were used: P_CU1-F1_ (TAAAAAATC GGTCGCTTCA), P_CU1-R1_ (GTCGTATTTTGCTTTCATAGCGGCCGCAAGCTTGTCATCGTCATCCTTGTAATCGATGTCATGATCTTTATAATCACCGTCATGGTCTTTGTAGTCCATTCCCCTTTTAGTCTTACACTT), P_CU1-F2_(AAGTGTAAGACTAAAAGGGGAATGGACTACAAAGACCATGACGGTGATTATAAAGATCATGACATCGATTACAAGGATGACGATGACAAGCTTGCGGCCGCTATGAAAGCAAAATACGAC) and P_CU1-R2_ (CTACAATGGCTCACATTGG). The resulting plasmid, pJB*legU1*, was then electroporated into the appropriate strains, selecting for Thy+.

### Mammalian Cell Culture and Transfection

HEK293T and HeLa cell lines were stored in our laboratory and cultured as previously described [22]. J774A.1 and Raw264.7 cells were grown at 37°C and 5% CO_2_ in Roswell Park Memorial Institute media (RPMI) (Gibco, USA) supplemented with 10% fetal bovine serum (Gibco, USA). For transfection, HEK293T and Hela cells were seeded and allowed to adhere overnight, followed by plasmid transfection using Lipofectamine 2000 reagent (Invitrogen, USA).

### Protein Localization in Transfected Mammalian Cells

Hela cells were seeded and transfected with the specified plasmids. To label 3 × Flag-tagged LegU1, the transfected cells underwent fixation with 4% paraformaldehyde (w/v), permeabilized using 0.1% Triton X-100 and subsequently immunolabeled. Staining was performed with a mouse anti-Flag primary antibody (Sigma-Aldrich F7425; 1:1000, USA), followed by an Alexa Fluor 488-conjugated anti-mouse secondary antibody (Invitrogen A-11029; 1:2000). To visualize the ER, Hela cells were stained with ER-Tracker (Thermo Fisher Scientific E34250, USA) as the manufacturer’s instruction. Image acquisition was conducted on a Zeiss LSM880 confocal microscope.

### Cell Fractionation

Cells were fractionated as previously described [23]. In brief, cells were harvested, washed in PBS medium, resuspended in MTE solution (270 mM D-mannitol, 10 mM Tris-base, 0.1 mM EDTA, pH 7.4) supplemented with protease inhibitor. The suspension was then lysed by sonication. After centrifugation of the cell lysates at 1,400 ×*g* for 10 min, a portion of the resulting supernatant was collected and designated as the whole-cell lysate fraction. The remaining supernatant was then submitted to centrifuge at 15,000 ×*g* for 10 min, after which the new supernatant was layered onto a continuous sucrose gradient (1–2 M). This gradient was subjected to ultra-centrifugation at 152,000 ×*g* for 70 min. A prominent band visible at the 1.3 M sucrose interface was aspirated and ultra-centrifuged again at 126,000 ×*g* for 45 min. The pellet was harvested as ER fraction and resuspended in PBS.

### Infection of Raw264.7

Raw264.7 macrophages was infected as described before [18]. Raw264.7 cells were plated at a density of 1 × 10^7^ cells per 100-mm dish. Cells were infected at an MOI of 150. Following inoculation, cells were subjected to centrifugation (500 ×*g*, 10 min) and subsequently maintained at 37°C for a further 50 min. Then the cells underwent four washes using PBS and further cultured in RPMI culture medium for 6 h. When indicated, 1 μg/ml of thapsigargin (Sigma-Aldrich), 10 μM MG132 (Sigma-Aldrich) or equal volume of DMSO solvent was introduced into the culture medium after the washes in PBS.

### Protein Purification

Expression of His-tagged BiP was induced in *E. coli* BL21 (DE3) cells through the addition of 0.1 mM of IPTG, followed by incubation at 20°C for 18 h. Bacterial cultures were collected and re-suspended in Lysis buffer [25 M Tris-HCl (pH 7.8), 500 mM NaCl, and 25 mM imidazole] containing 1% Triton, lysed by sonication. The cleared lysates were loaded onto an Ni Sepharose column (Sigma-Aldrich). The column was thoroughly washed using washing buffer [25 M Tris-HCl (pH 7.8), 500 mM NaCl, and 50 mM imidazole] with protease inhibitor (Thermo Fisherm Scientific), and eluted with Elution buffer [25 M Tris-HCl (pH 7.8), 500 mM NaCl, and 300 mM imidazole]. Finally, the eluted proteins were mixed with Preservation buffer [25 mM Tris-HCl (pH 7.8), 100 mM NaCl, 1 mM DTT], desalinated and enriched by 30 kDa ultrafiltration tube (Millipore, USA).

### Pull-Down Assay

*L. pneumophila* strains which harboring an empty vector or a vector encoding *N*-terminally 3 × Flag-tagged LegU1 was cultured in AYE at 37°C for 20 h. The cells were collected and resuspended in Lysis buffer with 1%Triton and protease inhibitor, followed by disruption through sonication. The cleared lysate was then incubated with Anti-Flag M2 affinity gel (Sigma-Aldrich) at 4°C for 18 h. After washing, the resin was incubated with purified His-tagged BiP for 6 h at 4°C. Unbounded proteins were removed by centrifugation and a series of washes. Proteins bind to the resin were released by boiling in sample buffer.

### Protein-Protein Docking

Prediction of the complex of LegU1 and BiP interaction according to the server proceeds in three steps: Input structures and options. The PDB files of LegU1 and BiP were obtained from UniProt (https://www.uniprot.org/) and uploaded to the initial submission page of Z-DOCK (v3.0.2). Selection of contacting residues. The next step was selection of contacting residues for each submitted protein, and we chose the entire amino acid sequence of each protein using default parameters. Viewing results. The model and detailed information of the docking result was downloaded and viewed through the interaction interface via PDBePISA (https://www.ebi.ac.uk/msd-srv/prot_int/). Visualization of the figure was obtained through PyMOL (v2.5.7).

### Immunoprecipitation

To test the interaction between LegU1 and BiP and the ubiquitination of BiP, HEK293T cells were transiently transfected with designated plasmids for 24 h. Subsequently, the cells were collected and lysed using IP lysis buffer containing 100 × protease inhibitor cocktail (Thermo Fisher Scientific). Where specified, transfected HEK293T cells were treated with 10 μM MG132 for 6 h in complete medium prior to lysis. The protein concentration of the cleared lysates was quantified with the BCA kit (Thermo Fisher Scientific). Lysates were diluted with additional lysis buffer to normalize protein concentrations to an equivalent amount per 1 ml volume. Anti-Flag M2 affinity gel (Sigma-Aldrich) was introduced into each normalized sample, and mixtures were agitated overnight at 4°C. For evaluation of ubiquitinated BiP in infection models, Raw264.7 cells infected with relevant *Legionella* strains were lysed and prepared as HEK293T cells. Immunoprecipitation was performed using anti-multiubiquitin agarose (MBL D058-8) according to the supplier’s guidelines. All of the immunoprecipitates were eluted by heating in sample buffer.

### *In vitro* Ubiquitination Assay

*In vitro* ubiquitination reactions were carried out following an established method [16]. In summary, HEK-293T cells were transfected with plasmids encoding 3 × FLAG-LegU1, 3 × FLAG-LegU1ΔF-box or an empty vector, along with untagged SKP1, CUL1, and RBX1. Twenty-four hours after transfection, cell lysates were collected and processed as described above for the immunoprecipitation protocol with anti-FLAG M2 resin (Sigma). The purified resin was mixed with 0.5 μg BiP, 25 ng UBA1 (E1), 0.25 μg Ubch5a (E2) , 2.5 μg ubiquitin, 1 × MgATP solution, and 0.5 μM ubiquitin aldehyde. The final reaction volume was brought to 10 μl using a buffer composed of 20 mM HEPES (pH 7.6), 1 mM DTT and 100 mM potassium acetate. The reaction was at 30°C for 90 min before terminating the process by heating in SDS sample buffer for 5 min. Resulting samples were then analyzed via immunoblotting. Enzymes, ubiquitin, and reaction buffers were supplied by Boston Biochem.

### Western Blot

Protein samples were resolved by SDS-PAGE and blotted no PVDF membranes (Millipore). For blocking, the membranes were treated with 5% skim milk at room temperature for one hour. Primary antibodies used were: anti-Calnexin (Proteintech Group 66903; 1:2000, China), anti-GFP (Proteintech Group 66002; 1:2000, China), anti-ICDH (a gift from Dr. Vogel JP; 1:2000), anti-BiP (Proteintech Group 11587; 1:2000), anti-CHOP (Proteintech Group 15204; 1:2000), anti-Actin (Proteintech Group 60008; 1:3000), anti-Tubulin (Proteintech Group 66240; 1:3000), anti-Flag (Sigma-Aldrich F7425; 1:2000), anti-HA (Sigma-Aldrich H9658; 1:2000), anti-Myc (Sigma-Aldrich M4439; 1:2000) , anti-His (Proteintech Group 66005; 1:2000).

### Statistical Analysis

Statistical analyses were conducted using SPSS software. Where applicable, Student’s t-test or ANOVA was applied using data from three independent biological replicates. Statistical significance, denoted by an asterisk (*) was defined as a *P*-value < 0.05.

## Results

### LegU1 Localizes to the ER in Eukaryotic Cells

**Fig. 1:**
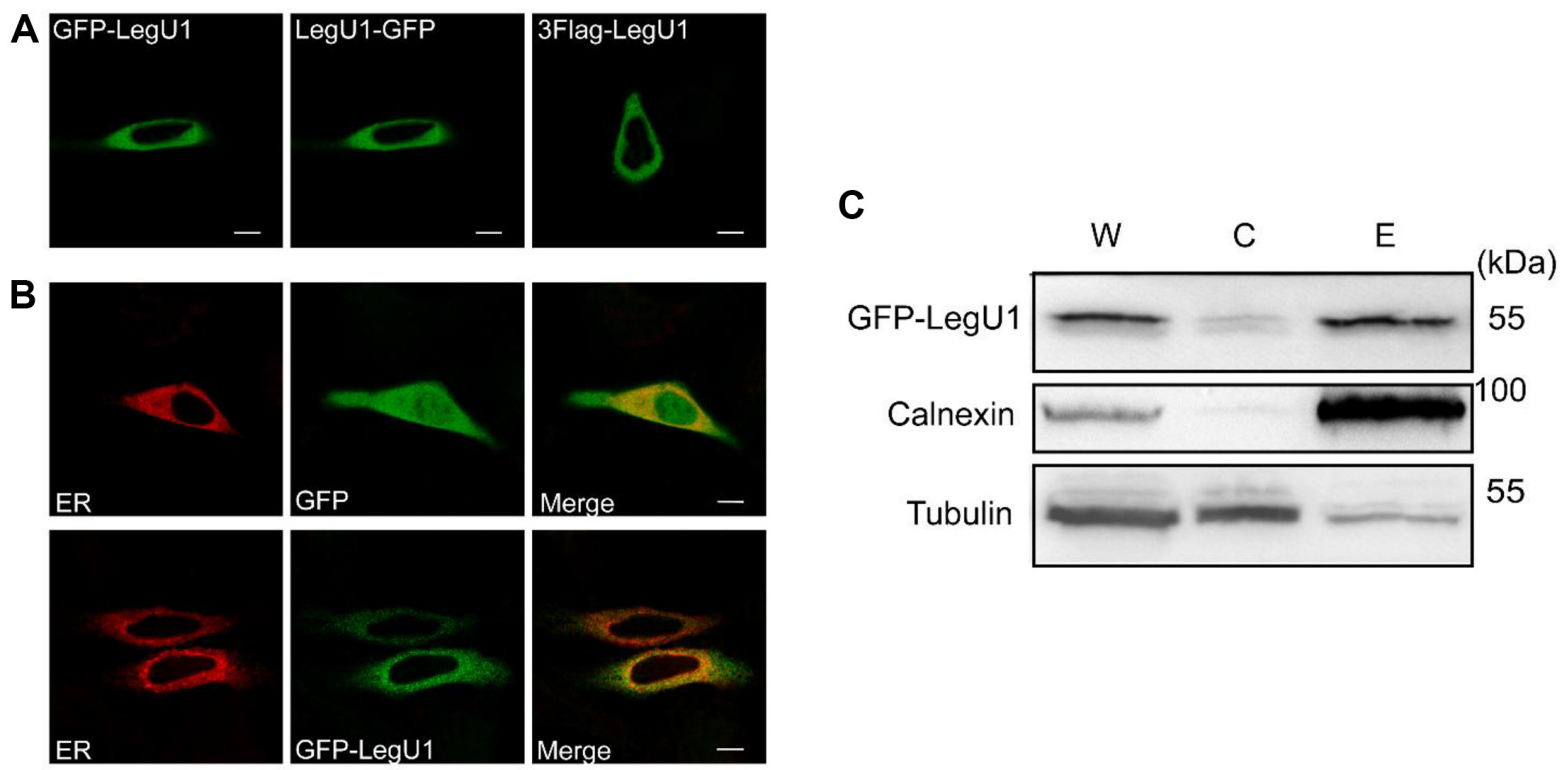
Ectopically expressed LegU1 is targeted to the ER in Hela cells. (**A**) Hela cells were transiently transfected with plasmids encoding the full-length LegU1 fused with N-terminal 3 × Flag tag, N-terminal or C-terminal GFP gene. The transfected cells were fixed with 4% (w/v) paraformaldehyde 24 h post-transfection, immunolabelled for Flag (right panel) and imaged by confocal microscopy. (**B**) Hela cells were transiently transfected with plasmids encoding the full-length or truncated mutants of LegU1 fused with GFP at the N-terminal (green) for 30 h before being stained with ER-Tracker (red) for observation by confocal microscopy. Scale bars, 10 μm. (**C**) cell fractionation and sucrose gradient ultracentrifugation experiments to analyze GFP-LegU1 localization in ER (E) and cytosol (C). Whole cell lysates (W) were loaded as a positive control. Although an equal amount of total protein was loaded in each lane, the Western blot data do not represent the relative amount of the GFPLegU1 protein in these cellular fractions because the relative levels of GFP-LegU1 in each cellular fraction are different.

We first determined the subcellular localization of LegU1 in eukaryotic cells (Fig. 1). To accomplish this, *legU1* was cloned into mammalian expression vectors by fusing to N-terminal of 3 × Flag epitope, N-terminal and C-terminal of the GFP gene. Next, the vectors were transfected into Hela cells for observation under confocal microscopy. The results showed that all of the fusion proteins were located in areas around the nucleus, indicating that the patterns of fusion do not affect the localization of LegU1, and suggesting that LegU1 targets to the ER (Fig. 1A). To validate this observation, we examined the localization of ectopically expressed GFP-LegU1 with ER marker (ER-Tracker). In contrast to GFP alone, GFP-LegU1 colocalized with the marker in Hela cells (Fig. 1B). To further verify the subcellular localization of LegU1, cell fractionation assays were performed. As shown in Fig. 1C, LegU1 was detected in the ER-enriched fraction, as confirmed by Western blot analysis using ER-specific marker calnexin. These results indicate that ectopically expressed LegU1 targets to the ER.

### The Hydrophobic Domains of LegU1 Are Required for ER Localization

**Fig. 2:**
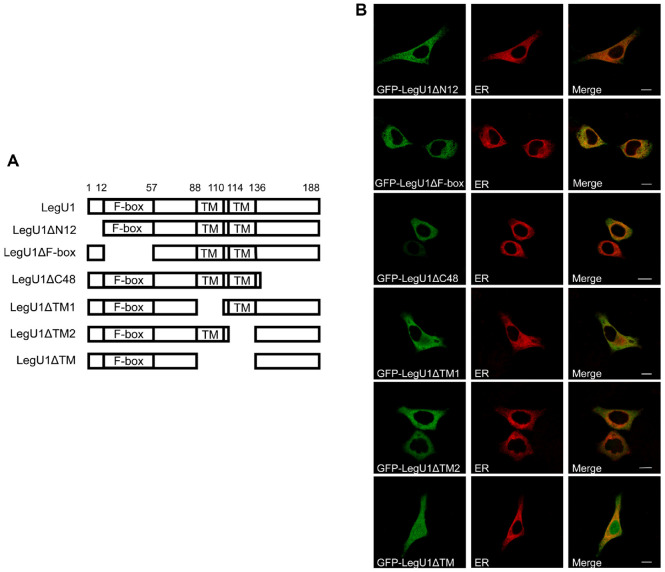
Residues 88-136 are essential for the localization of LegU1. (**A**) Schematic representation of LegU1 and the LegU1 mutants. TM, the predicted transmembrane domain. (**B**) Hela cells were transiently transfected with vectors encoding LegU1 or the indicated mutants fused with GFP at the N-terminal (green) and examined for colocalization by staining with ERTracker (red) 30 h post transfection. Scale bars, 10 μm.

Bioinformatic analysis revealed that LegU1 contains two hydrophobic transmembrane (TM) domains (the region from the 88th to the 136th residue, Fig. 2A). To determine whether the predicted transmembrane domains are essential for localization, a series of truncated *legU1* genes lacking a *N*-terminal domain (LegU1ΔN12), a F-box domain (LegU1ΔF-box), one of the TM domains (LegU1ΔTM1 or LegU1ΔTM2), both of the TM domains (LegU1ΔTM), or a C-terminal domain (LegU1ΔC48) were fused to *gfp* and expressed in Hela cells. As shown in Fig. 2B, GFP-LegU1ΔN12, GFP-LegU1ΔF-box, GFP-LegU1ΔC48, GFP-LegU1ΔTM1 and GFP-LegU1ΔTM2 localized to the ER, whereas LegU1ΔTM distributed diffusely throughout the cell, indicating that the predicted hydrophobic TM domains are required for the localization of LegU1 to the ER.

### Knockout of *legU1* Releases the Suppression of BiP during *L. pneumophila* Infection

**Fig. 3:**
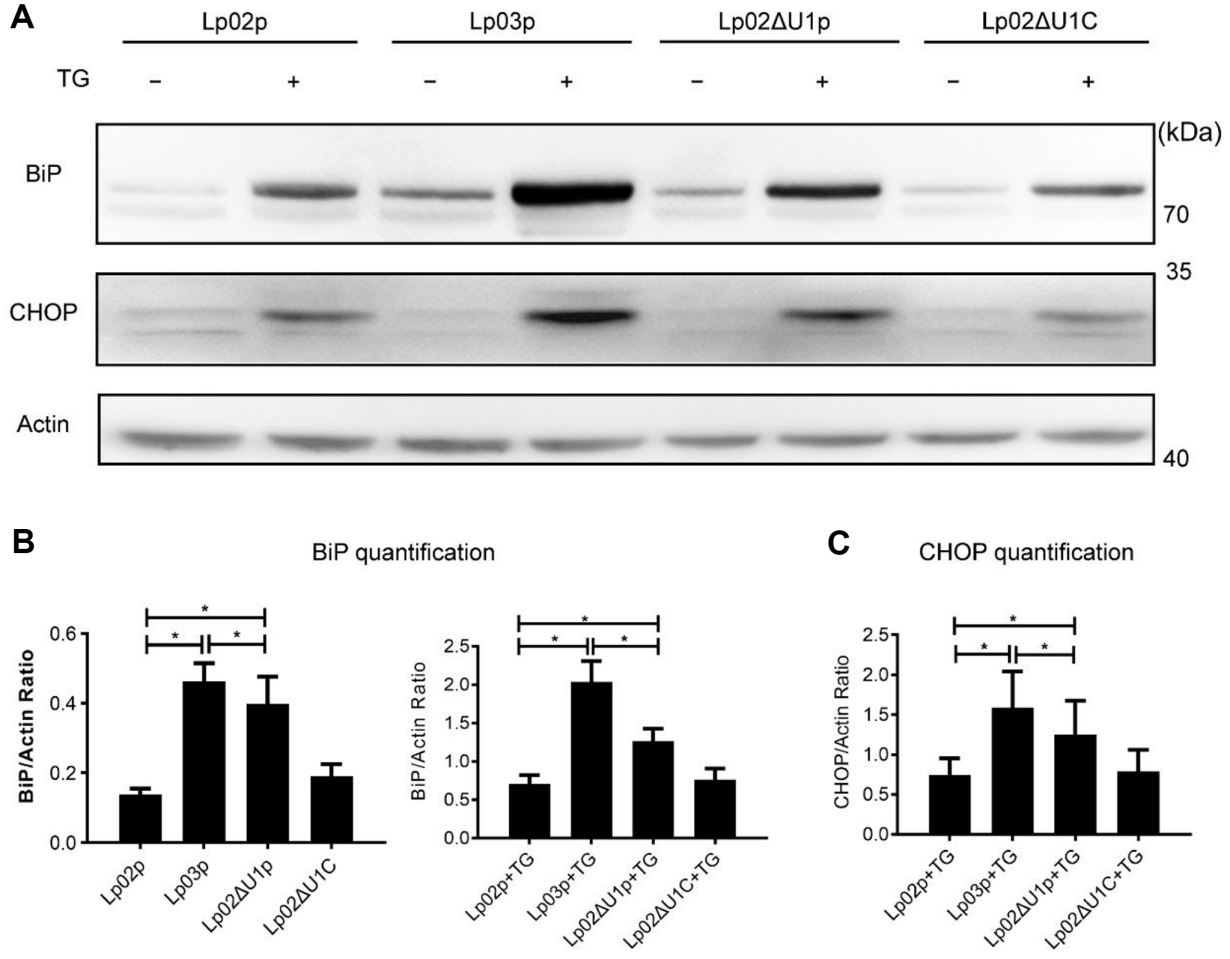
BiP and CHOP increase in the Δ*legU1* mutant strain-infected cells treated with thapsigargin. (**A**) Raw264.7 cells were either infected with wild type (Lp02p), *ΔdotA* (Lp03p), Δ*legU1* (Lp02ΔU1p) or *legU1* complemented (Lp02ΔU1C) strains of *L. pneumophila* at an MOI of 150. Cells were then untreated (−) or treated (+) with thapsigargin (TG; 1 μg/ml) for 6 h, after which the expression of BiP and CHOP was measured by immunoblotting. Actin was used as a loading control. (**B**) The levels of BiP in Raw264.7 cells were quantified from at least three biological replicates under the same experimental conditions as in (**A**). Data are depicted as the ratio of mean pixel intensity of BiP to that of Actin. (**C**) The levels of CHOP in Raw264.7 cells were quantified from at least three biological replicates under the same experimental conditions as in (**A**). Data are depicted as the ratio of mean pixel intensity of CHOP to that of Actin. Values in all graphs are the means ± s.e.m. *P < 0.05.

Previous study demonstrated that *L. pneumophila* can suppress the host UPR, and this suppression is dependent on the effector proteins secreted by T4BSS during infection [18]. Given that LegU1 localizes to the ER (Fig. 1) and involves in ubiquitination [16], which is important for regulating protein homeostasis in eukaryotic cells, we wondered whether LegU1 could facilitate suppression of the UPR by *L. pneumophila* during infection. To accomplish this, Raw264.7 cells were infected with avirulent *ΔdotA* (Lp03p), wild type (Lp02p), Δ*legU1* (Lp02ΔU1p), and *legU1* complemented (Lp02ΔU1C) strains and treated with or without thapsigargin (TG), a reagent that induces ER stress via depletion of luminal ER calcium stores, then two hallmarks of the UPR, the upregulation of BiP and the induction of pro-apoptosis factor CHOP [18], were tested. The results showed that compared to cells infected with Lp02p, the levels of BiP in cells infected with Lp02ΔU1p increased both with and without TG treatment. When LegU1 was expressed, BiP expression was comparable to that in the Lp02p control group (Fig. 3A and 3B). CHOP was only detected when infected cells were treated with TG, with a slight increase observed in the Δ*legU1* strain (Fig. 3A). These results suggest that LegU1 mutation results in an increase in BiP and CHOP. Interestingly, the amount of BiP or CHOP in Lp02ΔU1p-infected cells was lower than that in the Lp03p-infected cells (Fig. 3). We believe this is because there are other effectors that inhibit the UPR and participate in the suppression of BiP or CHOP during *L. pneumophila* infection, such as previously identified effectors that interfere with protein synthesis [18, 24, 25]. Taken together, these findings indicated that LegU1 contributes to UPR suppression during *L. pneumophila* infection.

### LegU1 Interacts Directly with BiP

**Fig. 4:**
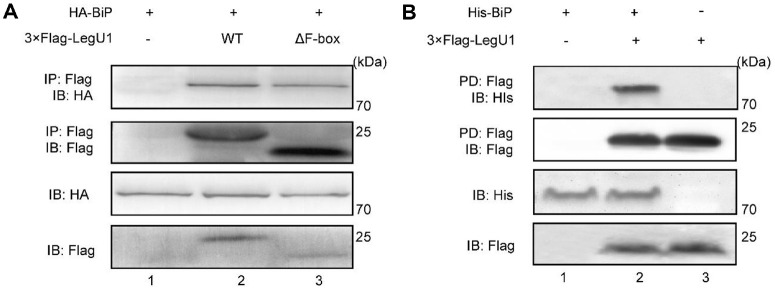
LegU1 interacts with BiP independently of the F-box domain. (**A**) co- (CoIP) of 3 × Flag-LegU1 and HA-BiP ectopically expressed in HEK293T cells. Whole cell extracts prepared from HEK293T cells transfected with the indicated plasmids were subjected to immunoprecipitation with anti-Flag antibody-conjugated beads followed by immunoblot analysis with anti-HA antibody. The expression level of each protein was monitored by immunoblot analysis with anti-HA and anti-Flag antibodies. (**B**) Pull-down (PD) assay shows direct interaction of LegU1 and BiP. Total cell lysate of *L. pneumophila* strains which harboring an empty vector or a vector encoding *N*-terminally 3 × Flag-tagged LegU1 was incubated with Anti-Flag M2 affinity gel. After washing for several times, the resin was then mixed with the purified His-tagged BiP. Proteins bind to the resin were released by boiling in sample buffer and analyzed by immunoblotting using anti-BiP antibody.

As LegU1 has been proved to confer an E3 ubiquitin ligase activity [16], the involvement of LegU1 in suppression of the upregulation of BiP and CHOP makes us wonder whether LegU1 could directly regulate them. Since CHOP could be hardly detected in cells infected with *L. pneumophila* alone (Fig. 3A), we focused our study on the relationship between LegU1 and BiP. We first conducted coimmunoprecipitation analysis to investigate whether LegU1 interacts with BiP. To test this possibility, HEK293T cells were co-transfected for 24 h with the plasmids expressing 3 × Flag-tagged LegU1 and HA-tagged BiP. Following cell lysis, proteins associated with LegU1 were isolated by immunoprecipitation and analyzed by immunoblotting. The results revealed that BiP was specifically enriched in the LegU1 immunoprecipitated relative to the vector control (Fig. 4A). Additionally, HA-tagged BiP was enriched in the cell lysate containing LegU1ΔFbox (Fig. 4A), indicating that the F-box domain of LegU1 is not essential for the interaction between LegU1 and BiP. Next, we examined the direct interaction of LegU1 and BiP by pull-down assay. Because LegU1 cannot be expressed in *E. coli* with unknown reasons, we utilized *L. pneumophila* to ectopically express LegU1 in our study (see the details in Methods). The immunoblotting results showed that only the resin incubated with the total lysate of *L. pneumophila* strain expressing 3 × Flag-tagged LegU1 could bind to BiP (Fig. 4B, Lane 2).

In many cases, because of the absence of an experimentally determined structure of the complex, protein-protein interactions can be modeled to obtain reliable understanding of their molecular basis. We used ZDOCK, a protein docking server, to predict interaction structure of LegU1-BiP complex. We found a positive protein affinity between LegU1 and BiP, implying that the interface surface can be interaction-specific (Fig. S1). Taken together, our results demonstrate that LegU1 directly interacts with BiP.

### LegU1 Mediates the Ubiquitination of BiP

**Fig. 5:**
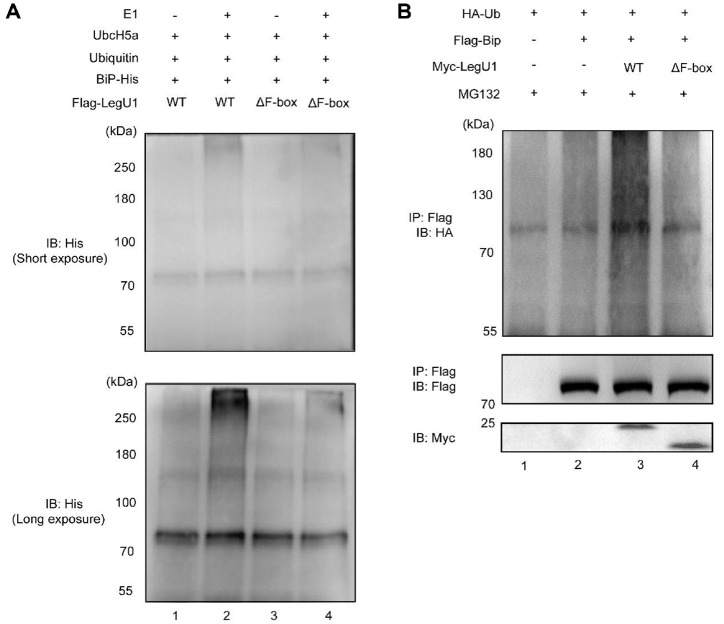
LegU1 directs the ubiquitination of BiP in a manner dependent on the F-box domain. (**A**) Resin harboring 3 × Flag-tagged LegU1 or LegU1ΔF-box was prepared by anti-FLAG immunoprecipitation from HEK-293T cells transfected with each translocated substrate. The resins were then used for *in vitro* ubiquitination experiments in a complete reaction mixture with wild-type ubiquitin, E1 enzyme, an E2 enzyme Ubch5a and BiP with C-terminal 6 × His tag. Immunoblot analysis was performed using anti-His antibody to detect ubiquitinated BiP. (**B**) The ubiquitination of BiP was measured in HEK293T cells transfected with the indicated combinations of plasmids. Whole-cell extracts prepared from the transfected cells were subjected to immunoprecipitation with anti-Flag antibody-conjugated beads. Immunoblot analysis was performed using anti-HA antibody in order to reveal ubiquitinated BiP. Then the membrane was stripped and re-probed with antibody directed against the Flag epitope. The expression of LegU1 and LegU1ΔF-box was monitored by immunoblot analysis with the anti-Myc antibody.

Previous studies showed that LegU1 interacts with Skp1 through the F-box domain to form SCF^LegU1^ complex and exhibits the activity of E3 ubiquitin ligase [16], the interaction between LegU1 and Skp1 was also confirmed in our study (data not shown). To further validate the interaction between LegU1 and BiP and, more importantly, to investigate whether LegU1 facilitates BiP ubiquitination, we examined BiP ubiquitination using an *in vitro* ubiquitination assay as before [16]. HEK293T cells were transfected to express 3×Flag-tagged LegU1, along with untagged Skp1, CUL1, and RBX1. The SCF^LegU1^ complex was subsequently purified via anti-Flag immunoprecipitation and then added to a mixture containing wild-type ubiquitin, E1 and E2 enzymes, and BiP with carboxy-terminal hexahistidine tag. After incubation, the mixtures were then subjected to Western blot to detect ubiquitinated BiP. The results showed that high-molecular-weight forms of BiP were specifically detected in LegU1 immunoprecipitate incubated with the E1 enzyme (Fig. 5A, lane 2). And the high-molecular-weight forms were not generated when the F-box domain of LegU1 was rendered nonfunctional (Fig. 5A, lane 4). These results show that LegU1 mediates the ubiquitination of BiP *in vitro*, and that BiP is a substrate of SCF^LegU1^ complex.

We then investigated whether LegU1 could mediate the ubiquitination of BiP in mammalian cells. For this purpose, plasmids encoding Myc-tagged LegU1, 3 × Flag-tagged BiP and HA-tagged ubiquitin were co-transfected into HEK293T cells. At 24 h after transfection, the cells were treated with MG132, a 26S proteasome inhibitor, to inhibit the proteasomal degradation of BiP. Next, BiP was concentrated using anti-Flag affinity beads and then analyzed for ubiquitination by Western blot using anti-HA antibody. Compared to cells expressing HA-ubiquitin only (Fig. 5B, lane 1), cells expressing both HA-ubiquitin and 3 × Flag-tagged BiP revealed more species of higher molecular weight BiP and stronger smearing (Fig. 5B, lanes 2, 3 and 4), suggesting that ectopically expressed BiP can ubiquitinated in HEK293T cells. Importantly, in contrast to cells that expressed BiP alone (Fig. 5B, lane 2), cells that concomitantly expressed LegU1 showed stronger smearing (Fig. 5B, lane 3), further indicating that LegU1 promotes BiP ubiquitination. Furthermore, the amounts of polyubiquitinated 3 × Flag-tagged BiP were decreased in lysate of cells producing 3 × Flag-tagged BiP and Myc-tagged LegU1ΔF-box, compared with lysates of cells producing 3 × Flag-tagged BiP and Myc-tagged LegU1 (Fig. 5B, lane 3, 4), demonstrating that the F-box domain of LegU1 is essential for the ubiquitination of BiP.

### LegU1 Modulates the Ubiquitination of BiP during *L. pneumophila* Infection

**Fig. 6:**
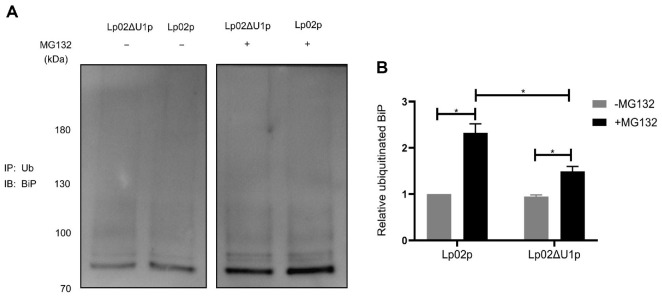
LegU1 facilitates the ubiquitination of BiP during *L. pneumophila* infection. (**A**) The ubiquitination of BiP was measured in Raw264.7 cells infected with the wild type (Lp02p) or Δ*legU1* (Lp02ΔU1p) strains. Raw264.7 cells were infected with the indicated strains of *L. pneumophila* at an MOI of 150. Cells were then untreated (−) or treated (+) with 10 μM of MG132 for 6 h. The cell lysates were then subjected to immunoprecipitation assay using anti-multiubiquitin antibodyconjugated beads, after which immunoblot analysis was performed using anti-BiP antibody. (**B**) The levels of ubiquitinated BiP in Raw264.7 cells were quantified from at least three biological replicates under the same experimental conditions as in (**A**). Data are depicted as the mean pixel intensity of ubiquitinated BiP in each sample. Values in all graphs are the means ± s.e.m. *P < 0.05.

To investigate whether LegU1 secreted by *L. pneumophila* during infection exerts the same activity on BiP as ectopically expressed LegU1 does, we analyzed the ubiquitination of endogenous BiP in Raw264.7 cells infected with the wild type (Lp02p) and Δ*legU1* (Lp02ΔU1p) strains of *L. pneumophila*. At 1 hpi, the infected cells were treated with or without MG132 for 6h. Then the cells were harvested, the lysates were immunoprecipitated with anti-multiubiquitin antibody-conjugated beads. Immunoprecipitation was then analyzed by Western blot using anti-BiP antibody. As shown in Fig. 6A and 6B, without treatment of MG132, the amount of ubiquitinated BiP from cells infected with Lp02p was similar to that from cells infected with Lp02ΔU1p. When the host cells were treated with MG132, the derivatives of ubiquitinated BiP were increased in both of the infected cells. Notably, the amount of ubiquitinated BiP in cells infected with Lp02p was significantly and statistically higher than that in cells infected with Lp02ΔU1p (Fig. 6A and 6B), indicating that LegU1 participates in the ubiquitination of BiP during *L. pneumophila* infection and ubiquitinated BiP could be degraded by the host proteasome.

## Discussion

*L. pneumophila* facilitates its intracellular survival and replication during infection by secreting multiple effector proteins that mimic the function of host enzymes involved in protein ubiquitination [15]. The *L. pneumophila* effector protein LegU1 has been proven to integrate into functional SCF complex that confer E3 ubiquitin ligase activity [16]. To date, the ubiquitination of host cell BiP during pathogen infection has been poorly characterized. In this work, we provided evidence that LegU1 mediates the ubiquitination of BiP, which is dependent on the F-box domain (Fig. 5), illustrating that BiP is the substrate of the SCF^LegU1^ E3 ligase. Our study provides further insights into the host-pathogen interaction network established by LegU1.

Studies have confirmed that BiP ubiquitination dose occur both *in vitro* and during infection. A multi-omics study revealed a comparative ubiquitination profile of A545-ACE2 cells infected with SARS-CoV-2 or SARS-CoV, which showed a significant increase in BiP ubiquitination 24 h after infection, however, the underlying mechanism remains unclear[26]. In eukaryotic cells, BiP has been shown to be ubiquitinated by multiple E3 ubiquitin ligases. For instance, the HECT-type E3 ligase NEDD4L was found to interact with BiP and ubiquitinate it at lysine 324 *in vitro*. Downregulation of BiP by NEDD4L has been shown to suppress both apoptosis and ER stress induced by acute liver injury [27]. An ER membrane-anchored E3 ubiquitin ligases GP78 and MUL1, a E3 ubiquitin ligases which is embedded in the outer mitochondrial outer membrane, also have been proved to regulate the amount of BiP through the ubiquitinational degradation [28, 29]. Both GP78 and MUL1 are RING finger E3 ligase, which bind to an E2 via its RING finger domain and to the substrates with the region distal to the RING finger. In the SCF complex, the RING finger protein RBX1, together with CUL1, forms the catalytic core that enables the recruitment of various F-box proteins (via SKP1) as substrate-recognition subunits, thereby conferring great diversity to the E3 ligase [9]. We speculate that LegU1 might mimic host cells E3 ubiquitin ligases, such as GP78 and MUL1, to downregulate BiP during *L. pneumophila* infection.

BiP has been traditionally regarded as an ER resident protein due to its C-terminal KDEL motif, which served as the ER retention signal [30]. However, ubiquitination primarily takes place in the cytosol, raising the question of how BiP becomes ubiquitinated. We acknowledge that a conceptual limitation of our study is the lack of direct biochemical evidence (*e.g.*, from protease protection or subcellular fractionation assays) to demonstrate the cytosolic localization of BiP during infection. Nevertheless, growing evidence show that BiP can also exists in different subcellular compartments outside the ER, such as cell surface, cytoplasm, nucleus, mitochondria, where it vastly expands its functional repertoire to deal with ER stress [31]. For example, Lee *et al*. reported that ER stress induces alternative splicing and alternative translation initiation of BiP, leading to the production of a cytosolic BiP isoform. Since this isoform lacks the signaling peptide but preserves the major functional domains of BiP, it localizes to the cytoplasm, where it can potentially interact with a variety of client proteins [32]. Additionally, BiP can undergo N-terminal arginylation mediated by arginyltransferase ATE1, which promotes its translocation to the cytosol under ER stress [33]. Furthermore, the observation that cytosolic, ER-associated, or mitochondrial E3 ubiquitin ligases, such as NEDD4, GP78, and MUL-1, mediate the ubiquitinated degradation of BiP under cellular stress supports the model that BiP can be relocalized from the ER and regulated via ubiquitination [27-29]. In this study, although direct proof of BiP's cytosolic localization is not yet available, our finding that LegU1 mediates ubiquitination of BiP (Fig. 5 and 6) and that deletion of *legU1* results in elevated BiP levels (Fig. 3) collectively support the conclusion that LegU1 mediates the ubiquitination of BiP. We propose that a subset of BiP is retro-translocated to the cytosol during *L. pneumophila*-induced ER stress, where it becomes accessible to the SCF^LegU1^ ubiquitination machinery. This model may also explain the relatively moderate ubiquitination phenotype observed during infection (Fig. 6).

As both an essential regulator and a target gene product of the UPR, the expression of BiP is increased in cases of ER stressors, and ER stress-dependent BiP up-regulation is usually operationally defined the UPR[34]. It has been reported that the wild type *L. pneumophila* infection prevents TG-induced upregulation of BiP at the protein level [18]. We show here that deletion of LegU1 impairs the suppression of BiP-upregulation (Fig. 3A and 3B) and LegU1 modulates the ubiquitination of BiP during *L. pneumophila* infection (Fig. 6), suggesting that ubiquitination-mediated degradation could be one strategy utilized by *L. pneumophila* to modulate BiP. It is also noteworthy that although the protein level of BiP in host cells infected with *legU1*-deleted strain is statistically higher than that infected with wild-type strain (Fig. 3A and 3B), the difference is just close to significant. This may be due to the reason that suppression of BiP is the result of synergistic effect of multiple effectors used by *L. pneumophila* to inhibit the UPR, such as LegK4 and other effectors (Lgt1, Lgt2, Lgt3, SidI and SidL) that have been known to inhibit protein synthesis [35, 36]. Previous study has reported that the macrophage infected by *L. pneumophila* without the 5 effectors (Lgt1, Lgt2, Lgt3, SidI and SidL) and LegK4 showed an upregulation of BiP after thapsigargin induction [24]. Therefore, a single LegU1 deletion is not sufficient to totally impair the ability of *L. pneumophila* to suppress the upregulation of BiP.

To date, several evidence suggests that bacterial pathogens utilize multiple strategies to deal with the UPR during infection [17]. However, BiP is rarely reported to be the target of bacterial pathogens. Previous studies showed that the effector VceC translocated by *Brucella abortus* interacts with host BiP for ER stress induction, promoting the infection of *B. abortus* [37]. Mhp271, a membrane protein of *Mycoplasma hyopneumoniae*, binds to BiP, mediates its downregulation, and inhibits the UPR, thereby facilitating bacterial adherence and infection [38]. Likewise, Shiga-toxigenic *Escherichia coli* (STEC) has been shown to secrete Subtilase cytotoxin (SubAB), an AB5 type toxin which could interact with and cleave BiP [39, 40]. Our finding that *L. pneumophila* T4BSS effector LegU1 mediates the ubiquitination of BiP further suggests the critical role of BiP in microbial pathogenesis. Targeting BiP may represent a key mechanism in *L. pneumophila* infection.

The UPR in eukaryotic cells consists of three branches of ER transmembrane sensors: activating transcription factor 6 (ATF6), inositol requiring kinase 1 (IRE1), and double-stranded RNA-activated protein kinase (PKR)-like endoplasmic reticulum kinase (PERK) [41]. Under normal conditions, BiP associates with these sensors and prevents their activation. Upon ER stress, BiP can detach from them, resulting the activation of them and the pathways they are responsible for [42, 43]. Previous studies showed that *L. pneumophila* inhibits IRE1 signaling by blocking XBP1 mRNA splicing, but induces the non-canonical processing and activation of ATF6. In addition, *L. pneumophila* infection alone has no effect on PERK activity [18, 25, 44]. To explore the relationship between LegU1 and the UPR, we examined the three sensors of the UPR. As shown in Fig. S2, deletion of LegU1 does not significantly affect ATF6 processing, blockage of XBP1 mRNA splicing and expression of PERK-mediated ATF4, suggesting that LegU1 does not participate in the suppression of the host UPR by modulating the activation of the UPR sensors. During the UPR, the upregulation of BiP is partly controlled by the ATF6 branch [44]. We propose that activation of ATF6 increases the expression of BiP during *L. pneumophila* infection, while LegU1 works as a regulator to control the protein level of BiP to a certain degree. This might help to inhibit the prolong activation of the UPR caused by excessive BiP accumulation and meanwhile maintain the homeostasis of the host cell. Future studies focusing on the role of the ATF6, IRE1 and PERK axis of the UPR during *Legionella* infection, as well as their underlying mechanisms, will improve our understanding of how LegU1 modulates the UPR.

Although both our work and previous studies proved that *L. pneumophila* inhibits the UPR during infection [18, 25], the function of inhibiting the UPR remains to be studied. Given that homeostatic activation of the UPR attempts to reduce the ER load and reestablish cell homeostasis, whereas maladaptive UPR outputs trigger apoptosis [41, 45], it is reasonable that manipulation of UPR may be advantageous for *L. pneumophila* to obtain the optimal condition for replication *in vivo*. Interestingly, deletion of LegU1 does not affect the intracellular proliferation of *Legionella* within amoebae and murine macrophages [16], indicating LegU1 and LegU1-mediated modulation of BiP have no direct effect on the multiplication of *L. pneumophila*. This corresponds to the existence of other strategies used by *Legionella* to compensate for the consequences of LegU1 deletion [35]. The use of multiple strategies makes the bacterium resilient in the presence of the UPR, which is consistent with the observation that *Legionella* could achieve robust intracellular replication even in host cells experiencing UPR [25]. Currently, we are focused on identifying the relationship between LegU1 and ER stress-induced apoptosis. Previous studies reported that, during *M. tuberculosis* (*Mtb*) infection, macrophages promote the massive expression and translocation of BiP through activation of the UPR, resulting in the translocation of Par-4 from the cytoplasm to the surface of the cell membrane, which leads to apoptosis in *Mtb*-infected macrophages, and thereby reducing the intracellular survival of *M. tuberculosis* [46]. This implies the important role of BiP in the ER stress-induced apoptosis. Thus, we wonder whether LegU1-medited regulation of BiP is associated with the ER stress-induced apoptosis. Given that LegU1 was found to facilitate the suppression of CHOP induction (Fig. 3A and 3C) and the critical role of CHOP pathway in ER stress-induced apoptosis [47], it’s possible that LegU1-mediated ubiquitination of BiP results in the suppression of CHOP, and thereby inhibit the UPR associated apoptosis.

## Supplemental Materials

Supplementary data for this paper are available on-line only at http://jmb.or.kr.
