# Supplementary material for: Legionella pneumophila Effector Protein LegU1 Mediates the Ubiquitination of Endoplasmic Reticulum Chaperone BiP
Source: J Microbiol Biotechnol. 2025 Oct 28;35:e2507056. doi: 10.4014/jmb.2507.07056 (PMC12602876; doi:10.4014/jmb.2507.07056)
Supplement: Supplementary file 1 [file jmb-35-e2507056-supple.pdf]

J. Microbiol. Biotechnol. 2025. 35: e2507056

https://doi.org/10.4014/jmb.2507.07056

Table S1. Bacterial strains and plasmids used in this study.

Strain or plasmid	Relevant properties	Reference or source
E. coli DH5α	host strain used for cloning	Lab collection
E. coli BL21 (DE3)	Host strain used for expression of his-tagged BiP	Lab collection
Lp02	Virulent *L. pneumophila* serogroup 1, strain Philadelphia, *rpsL, HsdR⁻, Thy⁻*	Lab collection
Lp02p	Lp02 containing pJB908	This study
Lp03	Virulent *L. pneumophila* serogroup 1, strain Philadelphia, *dot03*, *rpsL, HsdR⁻, Thy⁻*	[44]
Lp03p	Lp03 containing pJB908	This study
Lp02ΔU1	Lp02 with *legU1* deletion	This study
Lp02ΔU1p	Lp02ΔU1 containing pJB908	This study
Lp02ΔU1C	Lp02ΔU1 containing pJB*legU1* for complementation	This study
pBRDX	Suicide delivery vector, *rdxA sacB* Cm	[20]
pBR*ΔlegU1*	pBRDX::*legU1* for *legU1* deletion	This study
pJB908	pMMB66EH oriRSF1010 DoriT tdDi bla+	[21]
pJB*legU1*	LegU1 fusing to the N-terminal of 3 × Flag tag inserted in pJB908	This study
pET-28a (+)	Bacterial expression vector	Novagen
pcDNA3.1(+)	Mammalian expression vector	Invitrogen
pcDNA3.1(−)	Mammalian expression vector	Invitrogen
pIRES2-eGFP	Mammalian expression vector	Clontech
pIRES2-DsRed	Mammalian expression vector	Clontech

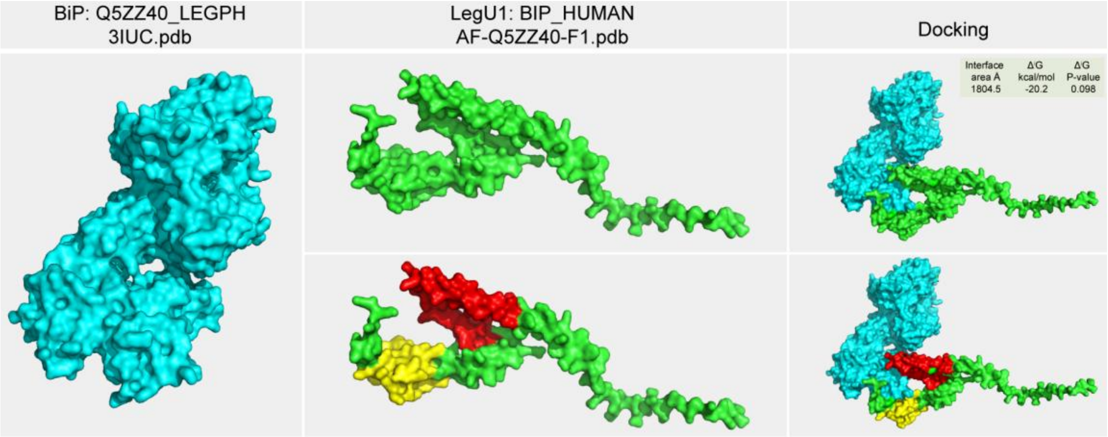

Fig. S1. **Model of LegU1–BiP interaction complex.** ZDOCK predicted possible binding modes in the translational and rotational space between the LegU1 and BiP and evaluates the pose using an energy-based scoring function. The red and yellow regions of LegU1 indicated the two hydrophobic transmembrane (TM) domains (the 88th to the 136th residue) and the F-box domain (the 12th to the 57th residue), respectively. The interface area in Å was 1804.5. $\Delta^{i}G$ was -20.2 kcal/mol, indicating the solvation free energy gain upon formation of the interface. Negative $\Delta^{i}G$ corresponds to positive protein affinity. $\Delta^{i}G$ P-value was 0.098. P<0.5 indicates interfaces with surprising (higher than would-be-average for given structures) hydrophobicity, implying that the interface surface can be interaction-specific.

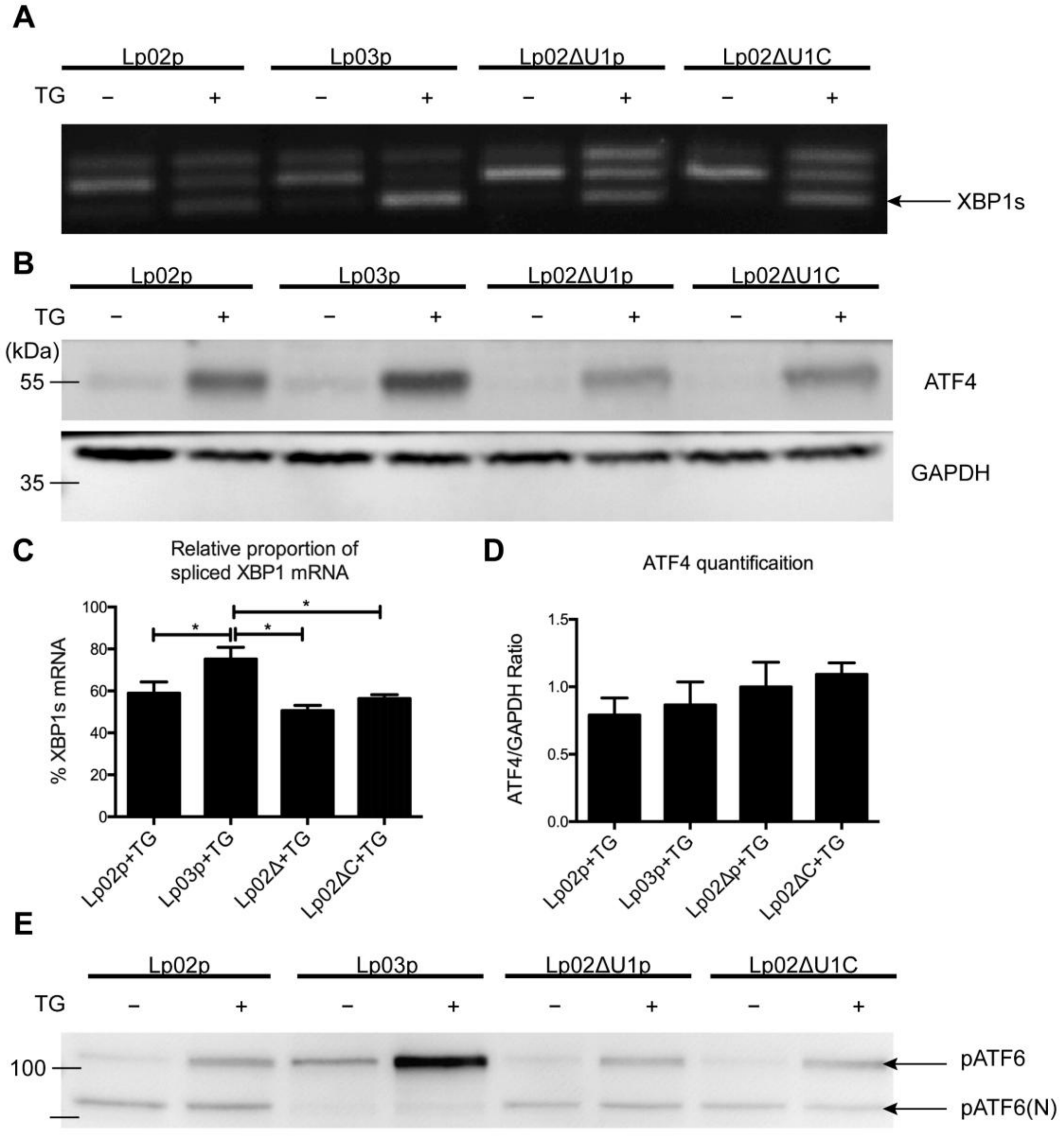

Fig. S2. **Deletion of LegU1 does not affect XBP1 mRNA splicing, ATF6 processing and ATF4 expression during *L. pneumophila* infection.** Raw264.7 cells were either infected with wild type (Lp02p), *ΔdotA* (Lp03p), *ΔlegU1* (Lp02ΔU1p) or *legU1* complemented (Lp02ΔU1C) strains of *L. pneumophila* at an MOI of 150. Cells were then untreated (−) or treated (+) with thapsigargin (TG; 1 μg/ml) for 6 h. The level of spliced XBP1 (XBP1s) mRNA was assayed via RT–PCR **(A)** and three biological replicates of XBP1 mRNA splicing during *L. pneumophila* infection and TG treatment were quantified (**C**). Values in all graphs are means ± s.e.m. *$P < 0.05$; Student's t-test. Expression of ATF4 were monitored via immunoblot (**B**). The level of ATF4 were quantified from at least three biological replicates and data are depicted as ratio of the mean pixel intensity of ATF4 to that of GAPDH (**D**). Full-length ATF6 (pATF6) and cleaved pATF6(N) were monitored via immunoblot (**E**).
